# Socio-economic inequalities in hypertension prevalence and care cascade in Bangladesh: Insights from a nationally representative survey

**DOI:** 10.1371/journal.pgph.0006712

**Published:** 2026-06-23

**Authors:** Md. Abdur Rafi, Urby Saraf Anika, Md. Golam Hossain

**Affiliations:** 1 Department of Public Health and Informatics, Bangladesh Medical University, Dhaka, Bangladesh; 2 Health Research Group, Department of Statistics, University of Rajshahi, Rajshahi, Bangladesh; Augusta University, UNITED STATES OF AMERICA

## Abstract

Hypertension is a leading contributor to non-communicable disease morbidity in Bangladesh. Despite increasing national attention to hypertension control, there remains limited population-level evidence quantifying how inequalities are distributed across the full cascade of diagnosis, treatment, and control using nationally representative data. We aimed to assess socioeconomic inequalities in hypertension prevalence, diagnosis, treatment, and blood pressure control. We analyzed data from the Bangladesh Demographic and Health Survey (BDHS) 2022, including 14,178 adults aged ≥18 years. Hypertension was defined as systolic blood pressure ≥140 mmHg, diastolic blood pressure ≥90 mmHg, or a previous diagnosis. Blood pressure control among treated individuals was defined as systolic blood pressure <140 mmHg and diastolic blood pressure <90 mmHg at survey measurement. Among participants with hypertension, we examined diagnosis, treatment, and control rates. Multivariable logistic regression estimated adjusted odds ratios (aORs) for factors associated with hypertension prevalence and the care cascade. Socioeconomic inequalities were assessed using the Relative Index of Inequality (RII), Slope Index of Inequality (SII), and Concentration Index (CnI). Overall, 22% of participants had hypertension (3,142/14,178), of whom 59% were previously diagnosed (1,863/3,142); among those diagnosed, 72.5% received treatment (1,350/1,863), and among those treated, 44% achieved blood pressure control (594/1,350). Older age (aOR 12.5, for ≥60 years), female sex (aOR 1.57), being in rich wealth quintile (aOR 1.20,), overweight (aOR 3.41), obesity (aOR 5.45), and type 2 diabetes mellitus (T2DM) (aOR 2.20) were associated with higher odds of hypertension. A pro-rich distribution of socioeconomic inequalities was evident in prevalence (RII 1.74, SII 0.28, CnI 0.09), diagnosis (RII 2.33, SII 0.42, CnI 0.17), treatment (RII 2.56, SII 0.39, CnI 0.15), and control (RII 1.50, SII 0.12, CnI: 0.07) of hypertension. Major gaps and socioeconomic inequalities persist in the hypertension care cascade in Bangladesh. Targeted, equity-focused strategies are essential for improving hypertension care.

## Introduction

Non-communicable diseases (NCDs) have emerged as the leading causes of morbidity and mortality worldwide. These chronic conditions now account for over 70% of global deaths, with a significant concentration of this burden in low- and middle-income countries (LMICs) [1]. Bangladesh, an LMIC in the south-east Asian region, is no exception to this global epidemiological transition. Over the past two decades, the country has experienced a shift in disease patterns, with over two-thirds of all deaths in recent years attributable to NCDs [2]. Among these, hypertension is one of the most prevalent and modifiable risk factors contributing to premature morbidity and mortality in the adult population [2,3]. Approximately one in every five adults in Bangladesh lives with hypertension [4].

However, despite this high prevalence, fewer than half of affected individuals are aware about their condition. Among those who are aware, majority reported receiving treatment; however, effective management remains inadequate. Blood pressure control rates are considerably lower compared to treatment uptake. Nearly, one-third of individuals receiving antihypertensive treatment achieve adequate blood pressure control [5,6]. This discrepancy between treatment and control highlights significant inefficiencies within the hypertension care cascade.

Socioeconomic inequalities further compound these gaps, leading to significant disparities in hypertension diagnosis, treatment, and control. Although the prevalence remains higher among socioeconomically advantaged groups, such as urban residents and those in higher wealth quintiles, recent evidence suggests a shifting distribution, with relatively faster increases now observed among socioeconomically disadvantaged populations [7]. These groups are disproportionately affected by the gaps in the care cascade [8,9]. Wealthier and urban residents typically have better access to healthcare services, including private facilities and specialized care, and are more likely to engage in preventive health practices. In contrast, socioeconomically disadvantaged populations face financial, geographic, and informational barriers to accessing timely and appropriate care [9].

As a signatory to the Sustainable Development Goals (SDGs), Bangladesh is committed to achieving these targets by 2030. Within SDG 3, reducing the burden of NCDs and attaining universal health coverage are major priorities. The rising prevalence of hypertension poses a significant challenge to achieving NCD-related targets, particularly the reduction of premature mortality. In addition, socioeconomic inequalities across the hypertension care cascade, such as diagnosis, treatment, and effective blood pressure control, represent barriers to universal health coverage by limiting equitable access to essential health services. Although a few previous studies in Bangladesh have examined sociodemographic differentials in hypertension prevalence and care cascade, most analyses remained descriptive and relied on earlier survey rounds of the Bangladesh Demographic and Health Survey (BDHS) [10,11]. A recent meta-analysis reported that individuals with higher educational attainment and richer wealth quintiles had a higher odds of hypertension [12]. While the 2022 BDHS report provides updated estimates of hypertension prevalence and care indicators, its reporting remains largely descriptive, offering limited insight into the magnitude and distribution of inequalities [13]. Furthermore, at the time this analysis was conducted, systematic quantification of these disparities using formal inequality measures was lacking using contemporary datasets. Few studies reported a pro-rich distribution of the hypertension prevalence and service utilizations like screening, diagnosis and treatment estimated from previous BDHS rounds [9,14]. In this context, a detailed assessment of socioeconomic inequalities is essential to generate evidence that can inform targeted, equity-oriented health policies and strategies.

Hence, the present study aimed to investigate the socioeconomic inequalities in the prevalence of hypertension and its care cascade in Bangladesh using nationally representative data from the 2022 Bangladesh Demographic and Health Survey (BDHS).

## Methods

### Data source

We conducted this analysis using data from the Bangladesh Demographic and Health Survey (BDHS), 2022 [15]. The BDHS employed a two-stage stratified cluster sampling design, ensuring coverage across both national and urban–rural strata. The sampling frame was based on the Integrated Multi-Purpose Sampling Master Sample developed by the Bangladesh Bureau of Statistics (BBS) using data from the 2011 population and housing census, which included enumeration areas (EAs) categorized by residence type.

In the first stage, 674 EAs were selected using probability proportional to size, stratified by administrative division and urban–rural location. A complete household listing was then conducted within each selected EA. In the second stage, approximately 45 households per EA were systematically sampled for interviews. A subset of one-third of these households was chosen for biomarker collection from children under five years and ever-married women aged 15–49 years. Additionally, in half of these biomarker-eligible households (representing one-sixth of all households), blood pressure and other biometric measurements were obtained from men aged 18 years and older, never-married women aged 18 years and older, and ever-married women aged 50 years and older.

Trained fieldworkers measured blood pressure using Multi-User Upper Arm Blood Pressure Monitors (UA-767F/FAC) equipped with medium cuffs, alongside UA-767PVS (small cuff) and UA-789AC (extra-large cuff) models to accommodate varying arm sizes. Each team was provided with three monitors and extra cuffs of each size. Measurements were taken from the left upper arm after the participant had rested in a seated position with the arm supported at heart level. Blood pressure was recorded three times at intervals of at least five minutes, and the average of the second and third readings was used to determine hypertension status [15].

The protocol for the 2022 BDHS received ethical clearance from both the ICF Institutional Review Board ethics committee and the Bangladesh Medical Research Council (BMRC). Informed written consent was obtained from each participant before commencement of the interview.

## Variables

### Outcome variables

Our primary outcome variable was the prevalence of hypertension, defined as having a systolic blood pressure greater than 140 mmHg and/or a diastolic pressure greater than 90 mmHg, based on the average of the second and third measurements, and/or a previous diagnosis of hypertension [15]. For the hypertension care cascade, we assessed three sequential stages: diagnosis, treatment, and blood pressure control. Participants with hypertension were considered diagnosed if they self-reported a prior medical diagnosis by a healthcare professional. Among those diagnosed, individuals were classified as receiving treatment if they reported taking prescribed antihypertensive medication within the 30 days preceding the survey. Finally, blood pressure control was defined as achieving a systolic blood pressure below 140 mmHg and a diastolic blood pressure below 90 mmHg at the time of measurement. Participants with readings above these thresholds were categorized as having uncontrolled hypertension [16].

### Independent variables

We structured the selection of explanatory variables based on Andersen’s Behavioral Model of health service use, which conceptualizes factors influencing healthcare utilization into three domains: predisposing, enabling, and need factors. This framework has been previously applied in chronic disease research within low- and middle-income countries [17,18]. In this study, predisposing factors included sex (male, female) and educational attainment (no formal education, primary, secondary, or higher secondary/above). Enabling factors comprised household wealth quintile (poorest, poorer, middle, richer, richest) and place of residence (urban, rural). The BDHS wealth index was derived through principal component analysis of household assets, amenities, and housing characteristics, subsequently categorized into quintiles. Though health insurance coverage is typically considered an enabling factor [17], this information was unavailable in the current dataset. Need factors included age (grouped in 10-year intervals), body mass index (BMI, categorized as underweight <18.5 kg/m², normal 18.5–24.9 kg/m², overweight 25–29.9 kg/m² and obese ≥30 kg/m²), and self-reported diagnosis of T2DM (yes, no).

## Statistical analysis

### Descriptive statistics

We summarized participant characteristics using frequencies and percentages for categorical variables and means with standard deviations for continuous variables. The prevalence of hypertension and proportions at each care cascade stage were estimated with corresponding 95% confidence intervals (CIs). To calculate the overall care cascade, we used the total hypertensive population as the denominator, calculating cumulative losses between each step, those undiagnosed, untreated, and uncontrolled relative to the initial hypertensive participants.

### Logistic regression models

To identify factors associated with hypertension prevalence and progression through the care cascade, we performed multivariable logistic regression models. Separate models were constructed for each outcome: diagnosis among hypertensive, treatment among those diagnosed, and blood pressure control among those treated. All independent variables, selected a priori based on Andersen’s model, were included in each model. Multicollinearity was assessed using the variance inflation factor (VIF), with values between 0 and 5 indicating no multicollinearity concerns. No multicollinearity issues were detected in our dataset. Model fit was assessed for all logistic regression models using the Hosmer-Lemeshow goodness-of-fit test, and all models demonstrated acceptable fit as indicated by non-significant test results (p > 0.05). Results were reported as adjusted odds ratios (aOR) with 95% CIs, and statistical significance was defined at a p-value threshold of <0.05.

### Socioeconomic inequalities

To assess socioeconomic inequalities in hypertension prevalence and care cascade, we used three complementary measures: the Concentration Index (CnI), the Slope Index of Inequality (SII), and the Relative Index of Inequality (RII). These indices describe both absolute and relative inequality across the full socioeconomic gradient, allowing a comprehensive evaluation [19]. The primary socioeconomic stratifier was household wealth status, derived from the BDHS wealth index, categorized into quintiles ranging from poorest to richest. Individuals were assigned a relative rank based on their position in the cumulative socioeconomic distribution. The SII estimated the absolute difference in hypertension prevalence and care cascade between the highest and lowest socioeconomic ranks, while the RII expressed this disparity as a relative ratio, comparing the likelihood of hypertension across the socioeconomic spectrum. The CnI quantified the extent to which hypertension prevalence and care cascade were concentrated among different socioeconomic groups, with positive values indicating higher prevalence among wealthier groups and negative values reflecting concentration among the poorer. Since our outcome variables were binary, we applied the Erreygers-corrected CnI to adjust for the bounded nature of the variable, ensuring meaningful comparisons. All inequality measures were calculated using individual-level data with 95% CIs, which were obtained using non-parametric bootstrap resampling with 1,000 iterations. All statistical analyses were conducted using R version 4.4.2. All analyses were conducted using the *‘survey’* package in R, incorporating BDHS sampling weights, primary sampling units, and stratification variables through complex survey design procedures to generate nationally representative estimates and valid standard errors in accordance with DHS analytical guidelines [20]. A detail of these analyses is provided in S1 File.

### Patient and Public Involvement

Patients and the public were not involved in the design, conduct, reporting, or dissemination plans of this research. This study utilized secondary data from a nationally representative survey, and therefore direct involvement of patients or community members was not feasible.

### Ethics approval and consent to participate

The protocol for the 2022 BDHS received ethical clearance from both the ICF Institutional Review Board ethics committee and the Bangladesh Medical Research Council (BMRC). Informed written consent was obtained from each participant before commencement of the interview.

## Results

### Sociodemographic characteristics

We included a total of 14,178 participants, with 34.5% from urban areas and 65.5% from rural. The mean age was 40.6 (SD 16.2) years with participants aged 18–40 years comprised 57% of the sample. Females accounted for 55% of participants. Approximately, 25% had no formal education, 25% completed primary, 33% secondary, and 16% higher secondary or above. Participants were almost evenly distributed across the wealth quintiles. The mean BMI was 23.7 kg/m² in urban participants and 22.3 kg/m² in rural. Overweight and obesity was present in 36% of urban participants versus 23% of rural. T2DM was diagnosed in 5.7% of the participants, with higher prevalence in urban areas (8%) than rural (4.4%) (Table 1).

**Table 1:** Sociodemographic characteristics of the participants (n = 14,178).

Characteristic	Overall, n = 14,178	Urban, n = 4,886	Rural, n = 9,292	p-value
Division				<0.001
Barishal	1,519 (10.71)	512 (10.48)	1,007 (10.84)	
Chattogram	1,984 (13.99)	824 (16.86)	1,160 (12.48)	
Dhaka	1,911 (13.48)	884 (18.09)	1,027 (11.05)	
Khulna	1,823 (12.86)	588 (12.03)	1,235 (13.29)	
Mymensingh	1,623 (11.45)	387 (7.92)	1,236 (13.30)	
Rajshahi	1,816 (12.81)	622 (12.73)	1,194 (12.85)	
Rangpur	1,768 (12.47)	510 (10.44)	1,258 (13.54)	
Sylhet	1,734 (12.23)	559 (11.44)	1,175 (12.65)	
Age (years), mean (SD)	40.56 (16.18)	39.43 (15.45)	41.15 (16.53)	<0.001
Age group (years)				<0.001
18-30	4,741 (33.44)	1,705 (34.90)	3,036 (32.67)	
31-40	3,294 (23.23)	1,197 (24.50)	2,097 (22.57)	
41-50	2,287 (16.13)	813 (16.64)	1,474 (15.86)	
51-60	2,000 (14.11)	648 (13.26)	1,352 (14.55)	
61-70	1,327 (9.36)	377 (7.72)	950 (10.22)	
>70	529 (3.73)	146 (2.99)	383 (4.12)	
Sex				0.657
Male	6,365 (44.89)	2,206 (45.15)	4,159 (44.76)	
Female	7,813 (55.11)	2,680 (54.85)	5,133 (55.24)	
Educational attainment				<0.001
No formal education	3,578 (25.24)	886 (18.13)	2,692 (28.97)	
Primary	3,571 (25.19)	1,079 (22.08)	2,492 (26.82)	
Secondary	4,708 (33.21)	1,688 (34.55)	3,020 (32.50)	
Higher secondary/above	2,321 (16.37)	1,233 (25.24)	1,088 (11.71)	
Wealth index				<0.001
Poorest	2,756 (19.44)	1,059 (21.67)	1,697 (18.26)	
Poor	2,733 (19.28)	823 (16.84)	1,910 (20.56)	
Middle	2,849 (20.09)	1,023 (20.94)	1,826 (19.65)	
Rich	2,856 (20.14)	1,049 (21.47)	1,807 (19.45)	
Richest	2,984 (21.05)	932 (19.07)	2,052 (22.08)	
BMI (kg/m^2^), mean (SD)	22.79 (4.20)	23.74 (4.38)	22.30 (4.02)	<0.001
BMI category				<0.001
Underweight (<18.5 kg/m^2^)	2,141 (15.10)	554 (11.34)	1,587 (17.08)	
Normal weight (18.5-24.9 kg/m^2^)	8,122 (57.29)	2,569 (52.58)	5,553 (59.76)	
Overweight (25-29.9 kg/m^2^)	3,200 (22.57)	1,390 (28.45)	1,810 (19.48)	
Obese (≥30 kg/m^2^)	715 (5.04)	373 (7.63)	342 (3.68)	
Diagnosed T2DM				<0.001
No	13,374 (94.33)	4,490 (91.90)	8,884 (95.61)	
Yes	804 (5.67)	396 (8.10)	408 (4.39)	

### Prevalence of hypertension

Overall, 22% of participants had hypertension. Prevalence increased with age, from 7% among those aged 18–30 years to 48% in participants over 70 years. Females had a higher prevalence (25%) than males (18%). Participants without formal education had the highest prevalence (30%), while those with higher secondary or above education had lower rates (19%). Hypertension prevalence was higher in urban (25%) than rural areas (21%). It also increased with wealth quintile, from 17% in the poorest to 27% in the richest group, and with BMI, affecting 13% of underweight and 44% of obese participants. Among those with diagnosed T2DM, 54% had hypertension (Table 2).

**Table 2:** Prevalence and care cascade of hypertension among the participants (n = 14,178).

Characteristic	Prevalence, % (95% CI) (N = 14,178)	Diagnosed, % (95% CI) (N = 3,142)	Treated, % (95% CI) (N = 1,863)	Controlled, % (95% CI) (N = 1,350)
Overall	22.16 (21.49 - 22.85)	59.29 (57.57 - 61)	72.46 (70.39 - 74.44)	44.22 (41.59 - 46.88)
Age group (years)				
18-30	6.77 (6.09 - 7.52)	42.06 (36.78 - 47.52)	44.44 (36.33 - 52.86)	51.67 (39.31 - 63.82)
31-40	18.03 (16.76 - 19.38)	49.49 (45.49 - 53.51)	62.24 (56.58 - 67.59)	53.01 (45.79 - 60.1)
41-50	27.98 (26.18 - 29.86)	61.25 (57.42 - 64.95)	69.9 (65.18 - 74.23)	41.24 (35.57 - 47.15)
51-60	37.9 (35.8 - 40.05)	66.89 (63.46 - 70.14)	78.3 (74.51 - 81.67)	46.6 (41.75 - 51.52)
61-70	43.26 (40.61 - 45.94)	66.03 (62.06 - 69.78)	83.11 (79.01 - 86.55)	41.27 (35.97 - 46.78)
>70	48.2 (43.97 - 52.46)	61.18 (55.07 - 66.95)	77.56 (70.4 - 83.4)	33.88 (26.06 - 42.7)
Sex				
Male	18.37 (17.43 - 19.34)	53.46 (50.6 - 56.31)	70.4 (66.71 - 73.85)	49.09 (44.45 - 53.75)
Female	25.25 (24.3 - 26.23)	62.75 (60.59 - 64.85)	73.51 (70.98 - 75.89)	41.87 (38.7 - 45.1)
Educational attainment				
No formal education	30.21 (28.73 - 31.74)	58.37 (55.41 - 61.28)	73.85 (70.29 - 77.13)	37.77 (33.48 - 42.25)
Primary	21.81 (20.49 - 23.2)	59.82 (56.34 - 63.21)	69.31 (64.98 - 73.33)	41.8 (36.54 - 47.24)
Secondary	17.91 (16.84 - 19.03)	59.91 (56.56 - 63.16)	71.29 (67.19 - 75.06)	47.5 (42.4 - 52.66)
Higher secondary/above	18.91 (17.37 - 20.56)	59.45 (54.8 - 63.94)	77.01 (71.54 - 81.7)	57.21 (50.3 - 63.86)
Residence				
Urban	24.91 (23.72 - 26.14)	63.43 (60.69 - 66.09)	75.78 (72.63 - 78.67)	46.15 (42.15 - 50.21)
Rural	20.72 (19.9 - 21.55)	56.68 (54.45 - 58.87)	70.12 (67.34 - 72.76)	42.75 (39.28 - 46.28)
Wealth index				
Poorest	17.34 (15.98 - 18.8)	53.56 (49.07 - 57.98)	61.72 (55.63 - 67.46)	43.04 (35.57 - 50.83)
Poor	19.39 (17.95 - 20.92)	53.02 (48.76 - 57.23)	68.68 (63.04 - 73.82)	39.9 (33.25 - 46.94)
Middle	22.25 (20.76 - 23.82)	57.57 (53.69 - 61.36)	68.49 (63.56 - 73.05)	39.2 (33.36 - 45.37)
Rich	24.23 (22.69 - 25.84)	61.42 (57.74 - 64.97)	76 (71.72 - 79.82)	46.75 (41.38 - 52.2)
Richest	27.08 (25.51 - 28.7)	66.34 (63.01 - 69.51)	79.48 (75.85 - 82.68)	47.65 (42.95 - 52.4)
BMI category				
Underweight	13.5 (12.12 - 15.01)	53.98 (48.22 - 59.64)	67.31 (59.61 - 74.18)	45.71 (36.51 - 55.23)
Normal weight	18.43 (17.6 - 19.29)	56.05 (53.52 - 58.54)	70.68 (67.51 - 73.66)	46.04 (42.06 - 50.06)
Overweight	32.47 (30.87 - 34.11)	63.14 (60.16 - 66.02)	72.56 (69.02 - 75.84)	42.23 (37.87 - 46.71)
Obese	44.34 (40.73 - 48)	66.88 (61.52 - 71.83)	83.02 (77.39 - 87.47)	42.61 (35.54 - 50)
Diagnosed T2DM				
No	20.23 (19.55 - 20.92)	55.75 (53.87 - 57.61)	70.29 (67.94 - 72.54)	43.21 (40.26 - 46.21)
Yes	54.35 (50.9 - 57.77)	81.24 (77.31 - 84.62)	81.69 (77.34 - 85.37)	47.93 (42.25 - 53.67)

### Care cascade of hypertension

Among participants with hypertension, 59% were previously diagnosed and it increased with age, from 42% in those aged 18–30 years to 67% in participants aged 51–60 years. A higher proportion of female participants (63%) were diagnosed compared to males (53%). Diagnosis rates were higher in urban participants (63%) than rural (57%). Across wealth quintiles, the proportion diagnosed ranged from 53% in the poorest to 66% in the richest. Around 81% of those with T2DM had previous diagnosis of hypertension also.

Among those diagnosed with hypertension, 72.5% received treatment and it increased with age. A total of 73% of female and 70% of male participants diagnosed with hypertension received treatment. Treatment rates ranged from 62% in the poorest to 79% in the richest participants. It was also higher among those who had obesity (83%) and T2DM (82%).

Among participants who received treatment, 44% achieved blood pressure control. Control rates were highest in participants aged 18–30 years (52%) and those with higher secondary or above education (57.2%). Despite higher treatment rates in participants with obesity and T2DM, control rates remained at 43% and 48%, respectively (Table 2).

**Fig 1:**
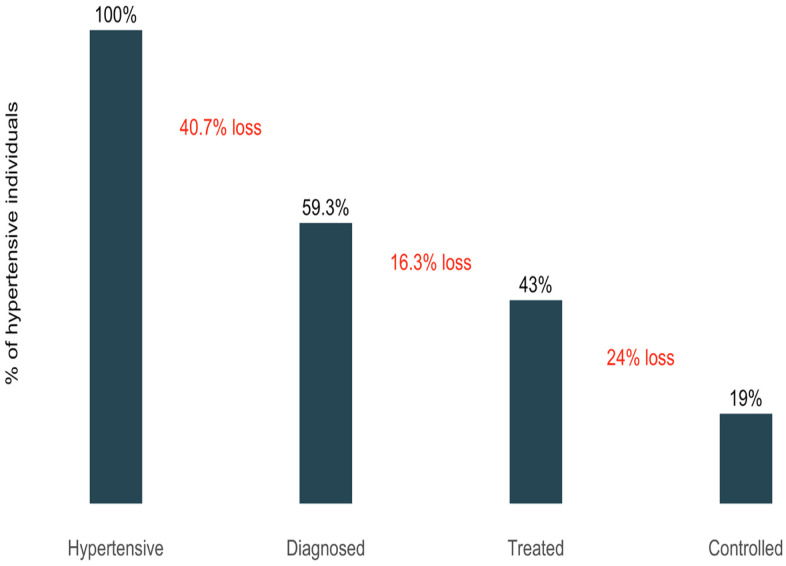
Care cascade among the participants with hypertension (n = 3,142).

Along the care cascade, 41% of participants with hypertension remained undiagnosed. An additional 16% were diagnosed but did not receive treatment, and 24% received treatment but could not achieve blood pressure control. Overall, 81% of all hypertensive participants either remained undiagnosed, untreated, or uncontrolled (Fig 1).

### Factors associated with prevalence and care cascade of hypertension

In multivariable analysis, age showed a strong, graded association with hypertension prevalence. Compared to participants aged 18–30 years, the aORs progressively increased in older groups (from aOR 2.70, 95% CI: 2.33–3.14 in 31–40 years to aOR 18.0, 95% CI: 14.3–22.7 in >70 years group). Female participants had higher odds of hypertension (aOR 1.57, 95% CI: 1.43–1.72) while rural participants had lower odds than urban (aOR 0.84, 95% CI: 0.77–0.93). Increasing wealth was associated with higher prevalence of hypertension, with significant associations in the middle (aOR 1.21, 95% CI: 1.05–1.40), rich (aOR 1.20, 95% CI: 1.04–1.40), and richest (aOR 1.24, 95% CI: 1.07–1.45) groups. Overweight (aOR 3.41, 95% CI: 2.89–4.02), obesity (aOR 5.45, 95% CI: 4.37–6.80), and having T2DM (aOR 2.20, 95% CI: 1.88–2.58) were also associated with higher odds of hypertension.

For diagnosis, higher odds were observed with increasing age, ranging from aOR 2.06, 95% CI: 1.55–2.75 in age group of 41–50 years to aOR 3.14, 95% CI: 2.30–4.30 in age group of 61–70 years. Female participants had higher odds of being diagnosed (aOR 1.56, 95% CI: 1.32–1.84) while rural residence was associated with lower odds of diagnosis (aOR 0.77, 95% CI: 0.65–0.91). Participants with primary (aOR 1.24, 95% CI: 1.01–1.52) and secondary education (aOR 1.36, 95% CI: 1.09–1.71) as well as belonging to the richest wealth quintile (aOR 1.38, 95% CI: 1.06–1.79) had higher odds of being diagnosed. Besides, those who had overweight (aOR 1.40, 95% CI: 1.05–1.87), obesity (aOR 1.52, 95% CI: 1.06–2.19), and T2DM (aOR 2.58, 95% CI: 2.00–3.37) had higher odds of diagnosis.

For treatment, participants had progressively higher odds with increasing age, ranging from aOR 2.13, 95% CI: 1.39–3.28 in 31–40 years to aOR 8.27, 95% CI: 5.14–13.4 in 61–70 years age group. Females (aOR 1.38, 95% CI: 1.09–1.76), those who were in richest wealth quintile (aOR 1.94, 95% CI: 1.34–2.81), and those who had obesity (aOR 2.81, 95% CI: 1.64–4.87) also had higher odds of receiving treatment while rural participants had a lower odds (aOR 0.72, 95% CI: 0.57–0.91).

For blood pressure control, participants aged >70 years (aOR 0.46, 95% CI: 0.23–0.90), those who had overweight (aOR 0.57, 95% CI: 0.36–0.90) or obesity (aOR 0.55, 95% CI: 0.32–0.94) were less likely to achieve control. Having higher secondary or above education was associated with better control of blood pressure (aOR 1.88, 95% CI: 1.25–2.84) (Table 3).

**Table 3:** Factors associated with prevalence and care cascade of hypertension among the participants (logistic regression models).

Characteristic	Prevalence, aOR (95% CI) (N = 14,178)	Diagnosis, aOR (95% CI) (N = 3,142)	Treatment, aOR (95% CI) (N = 1,863)	Control, aOR (95% CI) (N = 1,350)
Age group (years)				
18-30	Ref.	Ref.	Ref.	Ref.
31-40	2.70 (2.33, 3.14)*	1.27 (0.96, 1.69)	2.13 (1.39, 3.28)*	1.11 (0.61, 2.01)
41-50	4.91 (4.19, 5.75)*	2.06 (1.55, 2.75)*	2.97 (1.95, 4.55)*	0.66 (0.37, 1.18)
51-60	8.36 (7.11, 9.86)*	2.90 (2.17, 3.88)*	5.24 (3.41, 8.12)*	0.87 (0.49, 1.54)
61-70	12.5 (10.4, 14.9)*	3.14 (2.30, 4.30)*	8.27 (5.14, 13.4)*	0.67 (0.37, 1.20)
>70	18.0 (14.3, 22.7)*	2.87 (1.99, 4.18)*	6.27 (3.62, 11.0)*	0.46 (0.23, 0.90)*
Sex				
Male	Ref.	Ref.	Ref.	Ref.
Female	1.57 (1.43, 1.72)*	1.56 (1.32, 1.84)*	1.38 (1.09, 1.76)*	0.86 (0.67, 1.11)
Educational attainment				
No formal education	Ref.	Ref.	Ref.	Ref.
Primary	1.00 (0.88, 1.13)	1.24 (1.01, 1.52)*	1.02 (0.77, 1.37)	1.14 (0.85, 1.55)
Secondary	0.99 (0.87, 1.13)	1.36 (1.09, 1.71)*	1.22 (0.89, 1.68)	1.32 (0.96, 1.82)
Higher secondary/above	1.11 (0.93, 1.31)	1.22 (0.91, 1.64)	1.38 (0.91, 2.12)	1.88 (1.25, 2.84)*
Residence				
Urban	Ref.	Ref.	Ref.	Ref.
Rural	0.84 (0.77, 0.93)*	0.77 (0.65, 0.91)*	0.72 (0.57, 0.91)*	0.94 (0.74, 1.20)
Wealth index				
Poorest	Ref.	Ref.	Ref.	Ref.
Poor	1.06 (0.91, 1.23)	0.93 (0.71, 1.20)	1.30 (0.90, 1.90)	0.79 (0.51, 1.22)
Middle	1.21 (1.05, 1.40)*	1.06 (0.83, 1.36)	1.22 (0.86, 1.74)	0.80 (0.53, 1.21)
Rich	1.20 (1.04, 1.40)*	1.18 (0.91, 1.52)	1.66 (1.15, 2.39)*	1.04 (0.69, 1.55)
Richest	1.24 (1.07, 1.45)*	1.38 (1.06, 1.79)*	1.94 (1.34, 2.81)*	1.03 (0.69, 1.55)
BMI category				
Underweight	Ref.	Ref.	Ref.	Ref.
Normal weight	1.63 (1.41, 1.90)*	1.11 (0.85, 1.45)	1.39 (0.94, 2.05)	0.77 (0.50, 1.18)
Overweight	3.41 (2.89, 4.02)*	1.40 (1.05, 1.87)*	1.44 (0.95, 2.18)	0.57 (0.36, 0.90)
Obese	5.45 (4.37, 6.80)*	1.52 (1.06, 2.19)*	2.81 (1.64, 4.87)*	0.55 (0.32, 0.94)
Diagnosed T2DM				
No	Ref.	Ref.	Ref.	Ref.
Yes	2.20 (1.88, 2.58)*	2.58 (2.00, 3.37)*	1.34 (0.99, 1.83)	1.20 (0.91, 1.58)

Abbreviations: aOR: adjusted odds ratio; CI: confidence interval. * indicates statistical significance at p < 0.05.

### Socio-economic inequality in prevalence and care cascade of hypertension

We observed significant socio-economic inequalities in prevalence of hypertension. The overall RII was 1.74 (95% CI: 1.56–1.94), with males showing a higher level of inequality (RII 2.50, 95% CI: 2.08–3.02) compared to females (RII 1.38, 95% CI: 1.22–1.58). Urban and rural participants had comparable inequalities, with RIIs of 1.77 (95% CI: 1.49–2.10) and 1.74 (95% CI: 1.52–2.01), respectively. The corresponding SIIs for prevalence was 0.12 (95% CI: 0.10–0.15) overall, highest in males (0.17, 95% CI: 0.13–0.20) and urban participants (0.14, 95% CI: 0.10–0.19). The overall CnI for prevalence was 0.18 (95% CI: 0.14–0.21).

For diagnosis, inequalities persisted with an overall RII of 1.35 (95% CI: 1.21–1.51) and SII of 0.18 (95% CI: 0.12–0.24). Males showed higher inequality (RII 2.50, SII 0.17) than females (RII 1.38, SII 0.08). The overall CnI for diagnosis was 0.10 (95% CI: 0.06–0.13).

For treatment, the overall RII was 1.34 (95% CI: 1.21–1.49), with SII of 0.21 (95% CI: 0.14–0.29). Inequality was slightly higher in females (RII 1.38, SII 0.23) and urban participants (RII 1.43, SII 0.27). The overall CnI for treatment was 0.09 (95% CI: 0.06–0.13).

For control of blood pressure, socio-economic inequalities were modest. The overall RII was 1.26 (95% CI: 1.03–1.56), with an SII of 0.10 (95% CI: 0.01–0.20). Urban participants had greater inequality (RII 1.57, SII 0.21), while rural and sex-disaggregated estimates were non-significant. The overall CnI for control was 0.07 (95% CI: 0.01–0.14) (Table 4).

**Table 4:** Socio-economic inequality in prevalence and care cascade of hypertension among the participants.

Measure	Overall, Estimate (95% CI)	Male, Estimate (95% CI)	Female, Estimate (95% CI)	Urban, Estimate (95% CI)	Rural, Estimate (95% CI)
**Prevalence**					
RII	1.74 (1.56, 1.94)	2.50 (2.08, 3.02)	1.38 (1.22, 1.58)	1.77 (1.49, 2.10)	1.74 (1.52, 2.01)
SII	0.12 (0.10, 0.15)	0.17 (0.13, 0.20)	0.08 (0.05, 0.11)	0.14 (0.10, 0.19)	0.12 (0.09, 0.14)
CnI	0.18 (0.14, 0.21)	0.29 (0.23, 0.35)	0.10 (0.06, 0.15)	0.18 (0.13, 0.24)	0.18 (0.13, 0.22)
**Diagnosis**					
RII	1.35 (1.21, 1.51)	2.50 (2.08, 3.02)	1.38 (1.22, 1.58)	1.30 (1.12, 1.52)	1.74 (1.52, 2.01)
SII	0.18 (0.12, 0.25)	0.17 (0.13, 0.20)	0.08 (0.05, 0.11)	0.16 (0.07, 0.26)	0.12 (0.09, 0.14)
CnI	0.10 (0.06, 0.13)	0.29 (0.23, 0.35)	0.10 (0.06, 0.15)	0.08 (0.04, 0.13)	0.18 (0.13, 0.22)
**Treatment**					
RII	1.34 (1.21, 1.49)	1.30 (1.09, 1.58)	1.38 (1.23, 1.57)	1.43 (1.23, 1.66)	1.29 (1.13, 1.48)
SII	0.21 (0.14, 0.29)	0.19 (0.06, 0.32)	0.23 (0.15, 0.32)	0.27 (0.16, 0.38)	0.18 (0.09, 0.27)
CnI	0.09 (0.06, 0.13)	0.08 (0.03, 0.14)	0.10 (0.07, 0.14)	0.11 (0.07, 0.16)	0.08 (0.04, 0.12)
**Control**					
RII	1.26 (1.03, 1.56)	1.27 (0.90, 1.83)	1.22 (0.93, 1.61)	1.57 (1.18, 2.16)	1.08 (0.81, 1.49)
SII	0.10 (0.01, 0.20)	0.12 (-0.05, 0.28)	0.08 (-0.03, 0.20)	0.21 (0.08, 0.35)	0.03 (-0.09, 0.17)
CnI	0.07 (0.01, 0.14)	0.07 (-0.03, 0.19)	0.06 (-0.02, 0.15)	0.14 (0.05, 0.24)	0.02 (-0.07, 0.12)

Abbreviations: RII: relative index of inequality; SII: slope index of inequality; CnI = concentration index. Positive values of SII and CnI indicate concentration of the outcome among higher socioeconomic groups, while RII > 1 indicates higher relative risk among lower socioeconomic groups.

## Discussion

Overall, 22% our study participants had hypertension (25% of females and 18% of males); among them, just over half were aware of their condition, fewer than half received treatment, and only a small fraction achieved blood pressure control. We observed substantial socioeconomic inequalities in prevalence of hypertension and across the care cascade. While hypertension remained more prevalent among wealthier groups, those from poorer households had larger gaps in diagnosis, treatment, and blood pressure control.

Our observed prevalence of hypertension is consistent with prior national estimates indicating that approximately one-fifth of the adult population in Bangladesh is affected by this condition [4]. However, the BDHS 2017–18 reported a higher overall prevalence of 27%, with estimates of 28% among women and 26% among men [21]. Differences in sample composition, age distribution, and survey methodology between the two survey rounds might partly account for this discrepancy. Our findings suggest an improvement in awareness of hypertension diagnosis, with 59% of individuals with hypertension were aware of their diagnosis, compared to 42% in the 2017–18 BDHS [21] and 46% in the 2018 STEPS survey [5]. We found that 72.5% of those diagnosed with hypertension were receiving antihypertensive therapy, which is lower than the 87% reported by the BDHS 2017–18 [22], but higher than the 59% observed in the STEPS 2018 survey [5]. Approximately, 44% of individuals on treatment in our study achieved adequate blood pressure control, reflecting a modest improvement from the 34% reported in the BDHS 2017–18 [22] and the 40% reported in STEPS 2018 [5]. These findings suggest gradual progress in hypertension awareness and management in Bangladesh, although gaps in treatment coverage and blood pressure control still persist.

We observed that the prevalence of hypertension, as well as the rates of diagnosis and receiving treatment, progressively increased with advancing age, while the likelihood of achieving blood pressure control declined. This pattern closely mirrors trends documented in previous studies from the country [21,22]. Females had higher prevalence of hypertension and were more likely to be diagnosed and receive treatment, although this gender advantage did not translate into better blood pressure control outcomes, an observation also consistent with earlier reports [21–23]. One plausible explanation might be that women have greater contact with the healthcare system through reproductive and maternal health services, increasing opportunities for screening and diagnosis, whereas subsequent long-term management may be affected by lower treatment adherence or insufficient follow-up intensity. In addition, therapeutic inertia, inadequate supply of essential drugs at the provider level and limited structured chronic disease follow-up systems may contribute to suboptimal dose titration and long-term control among treated women. Besides, in our study, rural participants demonstrated significantly lower odds of hypertension prevalence, diagnosis, and receiving treatment. However, this contrasts with the findings of previous round of the BDHS, where no significant rural-urban differences were reported in these indicators [22,23]. This rural disadvantage in the hypertension care may reflect evolving inequalities in service availability, with increasing concentration of diagnostic and long-term management services in urban facilities, alongside persistent gaps in primary care readiness in rural areas [24]. Differences in health literacy, delayed care-seeking behavior, and limited continuity of care may further exacerbate these disparities. Higher educational attainment was positively associated with hypertension diagnosis in our study, but showed no significant association with receiving treatment or blood pressure control, aligning with prior findings [22]. Socioeconomic status, as measured by household wealth index, was similarly linked to higher prevalence, greater awareness, and increased likelihood of receiving treatment, though it remained unrelated to blood pressure control in our study as well as in previous reports [21–23]. Finally, participants with overweight, obesity, or T2DM had increased odds of being hypertensive, diagnosed, and treated, but a lower likelihood of achieving blood pressure control, patterns that corroborate existing evidence from earlier studies conducted in Bangladesh [21,22].

We found clear and consistent socio-economic inequalities in hypertension prevalence and its care cascade in Bangladesh. Overall, hypertension remained more concentrated among socioeconomically advantaged groups. For prevalence, the overall RII and positive SII indicated a higher concentration of hypertension among higher socioeconomic groups, with stronger relative inequality among males than females, suggesting greater socioeconomic stratification of risk in men. This pattern is reflective of early epidemiological transition, where modifiable risk factors such as diet, sedentary lifestyles, and stress initially predominate in wealthier, urban populations. Previous studies from Bangladesh also suggested this pattern [12,23]. However, recent reports suggest that the prevalence of hypertension has increased at a higher rate among socio-economically disadvantaged population of Bangladesh, making it a universal public health concern [4].

The inequality measures showed a consistent but heterogeneous pro-rich pattern across hypertension prevalence and the care cascade. Across the care cascade, relative inequalities in diagnosis and treatment were present but weaker (RII ~ 1.3–1.4) compared to prevalence, suggesting partial reduction in socioeconomic differentials once individuals enter the health system. In contrast, inequalities in blood pressure control were modest and less consistent, with lower RII and SII values, indicating relatively similar outcomes across socioeconomic groups among those treated. Rural–urban comparisons showed broadly comparable inequality patterns, suggesting that socioeconomic position rather than place of residence primarily drives disparities across cascade stages. Overall, inequalities were most pronounced in disease occurrence and attenuated in later care stages.

While prevalence of hypertension was higher among wealthier groups, individuals from socioeconomically disadvantaged households consistently faced greater gaps in diagnosis, treatment, and blood pressure control, consistent with the findings from previous studies [12,23,25–27]. These inequalities reflect structural barriers within the health system and broader social determinants of health, including unequal access to healthcare services, financial constraints, variable health literacy, and disparities in treatment adherence [28,29]. The socioeconomic inequalities persisted even among those receiving treatment, with blood pressure control rates remaining higher in affluent groups, particularly in urban areas. This indicates that inequalities are not limited to access alone but extend into the quality, continuity, and effectiveness of treatment. Urban health systems, although better resourced overall, often display greater internal inequalities. Socioeconomically disadvantaged urban residents may face barriers such as high out-of-pocket expenditure in private facilities, fragmented public services, and informal healthcare practices, resulting in poorer continuity of care and treatment adherence [30,31]. Conversely, rural areas, despite their limited infrastructure, showed fewer socioeconomic inequalities in hypertension control, likely due to uniformly constrained services that reduce the gradient between wealth groups. Such patterns of inequity in chronic disease management signal a need for the hypertension control programs of Bangladesh to incorporate equity-sensitive strategies that address not only service coverage but also the quality and outcomes of care across socioeconomic groups.

To address the escalating burden of hypertension and its complications, the Government of Bangladesh, in collaboration with the National Heart Foundation of Bangladesh and Resolve to Save Lives, introduced the Bangladesh Hypertension Control Initiative. Since its inception in 2018, the initiative has progressively implemented the World Health Organization (WHO) HEARTS technical package for hypertension management in public health facilities across the country [32]. The program emphasizes a simplified, evidence-based hypertension treatment protocol, ensures the uninterrupted availability of essential antihypertensive medicines free of charge, promotes team-based care involving both physicians and trained non-physician providers, and utilizes a simple digital hypertension management application for real-time data collection and patient monitoring. In 2020, a continuous quality improvement strategy was incorporated to reduce missed follow-up visits and enhance blood pressure control rates among patients under care [8]. Despite these policy-level interventions, we observed significant gap in the care cascade of hypertension. To close this gap, we would recommend a suite of equity-centered strategies. Community-based hypertension screening and case management should be expanded in underserved and hard-to-reach populations, particularly in rural and remote areas, through outreach clinics and deployment of community health workers. Primary healthcare capacity should be strengthened through sustained investment in diagnostic equipment, task-shifting training, and uninterrupted availability of essential antihypertensive medicines. In addition, financial protection mechanisms, including targeted subsidies or reduced-cost provision of antihypertensive drugs for low-income groups, should be considered to reduce treatment discontinuation due to cost barriers. At the programmatic level, national hypertension control programs should incorporate routine equity monitoring indicators, including: (i) hypertension detection rate by wealth quintile and geographic region, (ii) treatment initiation rate by socioeconomic group, (iii) blood pressure control rate stratified by sex, residence, and wealth status, and (iv) cascade drop-off between diagnosis, treatment, and control across strata. Routine disaggregated reporting of these indicators within national noncommunicable disease surveillance systems would enable continuous monitoring of inequities and support evidence-informed resource allocation over time.

Our study’s strengths include nationally representative data, objectively measured blood pressure, and comprehensive assessment of the care cascade using established inequality indices such as CnI, SII and RII. These provide a rigorous, multidimensional understanding of socioeconomic inequalities in hypertension care cascade in Bangladesh. However, our study had several limitations. First, we relied on self-reported data for hypertension diagnosis and treatment, which may be affected by recall or social desirability biases in diagnosis and treatment. Second, due to cross-sectional nature of the study, blood pressure control was measured at a single time point, which may not reflect true longitudinal control. Besides, the care cascade is dynamic, and individuals may transition between stages over time. This temporal fluidity cannot be captured in our study, potentially underestimating or overestimating the true progression through care. Third, wealth quintiles derived from household assets are well-established proxies for socioeconomic status, but they do not include aspects like employment stability, debt, or social capital. Finally, we lacked detailed data on major health system enablers, including health insurance coverage, physical distance to health facilities, provider density, and transportation costs. The absence of these variables may have led to residual confounding in our assessment of socioeconomic inequalities, potentially biasing both the magnitude and gradients of observed disparities in hypertension care. In addition, estimates for certain subgroups (particularly older age categories) showed wide confidence intervals, likely reflecting small sample sizes within these strata; therefore, these subgroup-specific findings should be interpreted with caution.

Moving forward, we need cohort studies integrating blood pressure monitoring, medication adherence, and evolving social determinants to clarify causal pathways. Additionally, qualitative studies including patients, providers, and communities would deepen contextual insights. Evaluating the effectiveness of targeted, equity-focused interventions, such as rural screening programs and community clinic-based drug distribution [33], is essential to inform scalable, evidence-based strategies for reducing hypertension care inequalities.

## Conclusion

We found persistent socioeconomic inequalities in hypertension prevalence, diagnosis, treatment, and control in Bangladesh. Despite higher prevalence among the wealthier groups, those from poorer households bear a disproportionate burden of undiagnosed, untreated, and uncontrolled hypertension. Bridging these inequalities is essential to ensure equity and inclusion for achieving universal health coverage. National hypertension control programs should be strengthened with explicit equity-focused policies and interventions that prioritize the needs of disadvantaged communities, with a particular focus on closing diagnosis and treatment gaps among the poorest wealth quintiles. In addition, routine monitoring of equity indicators across the hypertension care cascade should be established as a core performance metric to track progress over time and guide equitable program implementation in Bangladesh.

## Supporting information

S1 File Measurement of socioeconomic inequality in hypertension. (DOCX)
