# Supplementary material for: Socio-economic inequalities in hypertension prevalence and care cascade in Bangladesh: Insights from a nationally representative survey
Source: PLOS Glob Public Health. 2026 Jun 23;6(6):e0006712. doi: 10.1371/journal.pgph.0006712 (PMC13289857; doi:10.1371/journal.pgph.0006712)
Supplement: S1 File — (DOCX) [file pgph.0006712.s001.docx]

# **Measurement of Socioeconomic Inequality in Hypertension**

# To quantify socioeconomic inequalities in hypertension prevalence and care cascade, we applied three complementary measures of health inequality: the Concentration Index (CnI), Slope Index of Inequality (SII), and Relative Index of Inequality (RII). These measures demonstrate different aspects of inequality, relative versus absolute, overall distributional differences, and allow for comprehensive interpretation of disparities across the entire socioeconomic gradient.

## **Socioeconomic Ranking Variable**

Our primary socioeconomic stratifier was the household wealth quintile variable (*hv270a*), categorized into five groups from 1 (poorest) to 5 (richest). Each individual was assigned a relative fractional rank $R_{i}$ based on the cumulative distribution of the population sorted by wealth quintile. The fractional rank is calculated as:

$$R_{i}=\frac{i-0.5}{N}$$

where $i$ is the individual’s position in the ranked socioeconomic distribution (from poorest to richest) and $N$ is the total sample size. This fractional rank places individuals on a continuous scale from 0 (lowest socioeconomic status) to 1 (highest socioeconomic status), enabling inequality measures to reflect the entire socioeconomic distribution rather than just comparing extreme groups.

## **Concentration Index (CnI)**

The Concentration Index (CnI) is a widely used summary measure of socioeconomic inequality in health that quantifies the extent to which a health variable is distributed unequally across the socioeconomic spectrum.

The CnI for an individual-level binary health outcome $h_{i}$ (e.g., hypertension status: 1 if hypertensive, 0 otherwise) with mean $\mu$ is mathematically expressed as:

$$C=\frac{2}{\mu} \text{Cov}\left( h_{i},R_{i} \right)$$

where $\text{Cov}\left( h_{i},R_{i} \right)$ denotes the covariance between the health outcome and the fractional socioeconomic rank. The concentration index ranges from -1 to +1:

- A value of 0 indicates perfect equality (no socioeconomic inequality).
- A negative value indicates the health outcome is concentrated among the poor (pro-poor inequality).
- A positive value indicates concentration among the rich (pro-rich inequality).

### **Erreygers Correction for Binary Outcomes**

Since hypertension is a binary variable bounded between 0 and 1, the classical CnI can be biased, with its range depending on the mean prevalence. To address this, the Erreygers Corrected Concentration Index (ECCI) was applied, which adjusts the classical CnI to provide an inequality measure with a consistent interpretation regardless of outcome prevalence.

The ECCI is defined as:

$$E=\frac{4\mu}{b-a}\times C=\frac{4\mu}{1-0}\times C=4\mu C$$

where $a$ and $b$ are the lower and upper bounds of the health variable (0 and 1 for binary), and $\mu$ is the mean of the health variable.

The ECCI corrects for the bounded nature of binary variables, providing an index that varies between -1 and 1, facilitating meaningful comparisons across groups and over time.

### **Estimation and Confidence Intervals**

The concentration index and its Erreygers correction were estimated using individual-level data. The covariance term was calculated between hypertension status and fractional socioeconomic rank. To obtain robust 95% confidence intervals, bootstrap resampling with 1000 iterations was performed, sampling with replacement from the original dataset and recalculating the ECCI in each bootstrap sample.

## **Slope Index of Inequality (SII)**

The Slope Index of Inequality (SII) is a regression-based measure that estimates the absolute difference in the health outcome between the extremes of the socioeconomic hierarchy, accounting for the entire distribution and the relative size of each socioeconomic group.

The SII uses a continuous socioeconomic rank variable $R_{j}$, representing the midpoint cumulative population rank of each group, to reflect socioeconomic position on a relative scale from 0 (lowest) to 1 (highest). This approach accounts for the relative population share and allows for a more nuanced estimate of inequality across all groups.

The SII is estimated via linear regression of the health outcome on the socioeconomic rank:

$$h_{j}=\alpha+\beta R_{j}+\varepsilon_{j}$$

where:

- $h_{j}$ is the mean hypertension prevalence in socioeconomic group $j$,
- $R_{j}$ is the relative rank of group $j$,
- $\beta$ is the estimated slope coefficient representing the SII.

The SII ($\beta$) is interpreted as the absolute percentage point difference in hypertension prevalence between the lowest and highest socioeconomic groups, adjusting for the distribution of groups.

The SII was calculated using individual-level data by assigning each individual the relative rank of their socioeconomic group and fitting a linear regression model with hypertension status as the dependent variable and the fractional rank as the independent variable.

To obtain robust 95% confidence intervals for the SII, we applied bootstrap resampling with 1000 iterations, recalculating the SII in each sample.

## **Relative Index of Inequality (RII)**

The Relative Index of Inequality (RII) complements the SII by quantifying the relative difference in the health outcome across the socioeconomic spectrum, expressed as a ratio rather than an absolute difference.

Like the SII, the RII uses the fractional socioeconomic rank variable $R_{j}$, but estimates inequality through a generalized linear model with a log link function:

$$\log\left( h_{j} \right)=\alpha+\gamma R_{j}+\varepsilon_{j}$$

where $h_{j}$ is the expected hypertension prevalence in group $j$, and $\gamma$ is the regression coefficient.

Exponentiating $\gamma$ yields the RII:

$$RII=e^{\gamma}$$

interpreted as the ratio of hypertension prevalence at the highest socioeconomic rank (richest) to that at the lowest rank (poorest), accounting for the entire distribution.

An RII of 1 indicates no relative inequality; values greater than 1 indicate higher prevalence among the rich; less than 1 indicates higher prevalence among the poor.

### **Choice of Regression Model: Poisson vs. Log-binomial**

For estimation, we employed Poisson regression with robust variance estimation to model the binary hypertension outcome with a log link function.

While log-binomial regression is the direct method to estimate relative risks for binary outcomes, it often suffers from convergence issues and instability, especially when the outcome is common or sample sizes are limited. Poisson regression with robust standard errors provides a practical alternative that yields consistent and reliable estimates of relative risks and their confidence intervals without convergence problems.

### **Estimation and Confidence Intervals**

We fit a Poisson regression model with hypertension status as the outcome and the socioeconomic fractional rank as the independent variable. The exponentiated coefficient of the fractional rank variable provided the RII estimate. Bootstrap resampling with 1000 iterations was used to generate robust 95% confidence intervals.
