# Supplementary material for: Socio-economic inequalities in hypertension prevalence and care cascade in Bangladesh: Insights from a nationally representative survey
Source: PLOS Glob Public Health. 2026 Jun 23;6(6):e0006712. doi: 10.1371/journal.pgph.0006712 (PMC13289857; doi:10.1371/journal.pgph.0006712)
Supplement: Attachment — Submitted filename: Review comments.docx [file pgph.0006712.s003.docx]

**Point-by-point response**

Date: May 19, 2026

Dear Editor,

We would like to thank you for your consideration of the manuscript “Socio-economic inequalities in hypertension prevalence and care cascade in Bangladesh: insights from a nationally representative survey" and for the feedback provided by the reviewers. We have now addressed the minor comments, and hope the paper is now deemed acceptable for publication in Contraception and Reproductive Medicine (Submission ID: PGPH-D-25-02335).

We have modified and revised the manuscript accordingly; detailed point–by–point corrections are given below:

**Editor Comments:**

Hypertension care cascade, especially using DHS data, is a widely studied topic. Previous studies have shown socioeconomic differences in hypertension prevalence (Hossain et al., 2025; Iqbal et al., 2021) and care cascade in Bangladesh (Babagoli et al., 2025; Ishaq & Kakoly, 2025; Rahman et al., 2015). All these studies reported socioeconomic inequities in hypertension care in Bangladesh. As such, what this study is adding to our understanding? Please carefully motivate your work. Using new data (i.e., 2022 DHS) to report already known results may not be acceptable.

Also, hypertension prevalence by sociodemographic groups are clearly reported in the 2022 BDHS report Tables 14.3.1 (for women) and 14.3.2 (for men). From these table we clearly observe that prevalence of hypertension is higher among adults who are older, who have BMI at overweight/obese level, and who are from richest households. As such, what new information are you providing in your analysis that is not previous reported and not stated in the 2022 BDHS report? Note that obesity and diabetes are known risk factors of hypertension (<https://www.cdc.gov/high-blood-pressure/risk-factors/index.html>). The odds of hypertension should by higher among diabetic individuals and individuals with obesity. Hence, there is nothing new in reporting the known facts.

Hypertension care cascade by sociodemographic groups are reported in the 2022 BDHS report Tables 14.2.1 (for women) and 14.2.2 (for men). Estimates reported in these tables clearly shows that hypertension diagnosis was significantly lower in poorer households compared to richer households. Similarly, likelihood of receiving treatment was also relatively lower in poorer households. As such, the socioeconomic inequity has already been documented in previous studies and the 2022 BDHS report. Hence, what is the contribution of the current study?

Please provide convincing arguments that your study is novel, not reporting already known facts, and provide new information which is critical for hypertension management in Bangladesh. The paper may not be considered for publication without substantial evidence of new knowledge presented.

On a technical note, analyzing DHS data requires use of appropriate survey weights entailing sampling frames ([https://dhsprogram.com/data/Guide-to-DHS-Statistics/Analyzing_DHS_ Data.htm](https://dhsprogram.com/data/Guide-to-DHS-Statistics/Analyzing_DHS_%20Data.htm)). It is not clear whether this has been followed while conducting statistical analysis. Please make sure that the estimates and 95% confidence intervals are obtained as per the DHS data analysis recommendations.

References

- Hossain, K., Shuvo, T. A., Hosna, A. U., & Dey, D. R. (2025). The Impact of Socioeconomic Inequalities on the Risk of Hypertension in Bangladesh: A Systematic Review and Meta‐Analysis. The Journal of Clinical Hypertension, 27(1), e14957.

- Iqbal, A., Ahsan, K. Z., Jamil, K., Haider, M. M., Khan, S. H., Chakraborty, N., & Streatfield, P. K. (2021). Demographic, socioeconomic, and biological correlates of hypertension in an adult population: evidence from the Bangladesh demographic and health survey 2017–18. BMC Public Health, 21(1), 1229.

- Babagoli, M. A., Chen, Y. H., Chakma, N., Islam, M. S., Naheed, A., & Boettiger, D. C. (2023). Association of socio-demographic characteristics with hypertension awareness, treatment, and control in Bangladesh. Journal of Human Hypertension, 37(11), 993-999.

- Ishaq, F., & Kakoly, I. J. (2025). Decomposition of socioeconomic inequalities of hypertension care utilization: Bangladesh experience. BMC Health Services Research, 25(1), 1486.

- Rahman, M. M., Gilmour, S., Akter, S., Abe, S. K., Saito, E., & Shibuya, K. (2015). Prevalence and control of hypertension in Bangladesh: a multilevel analysis of a nationwide population-based survey. Journal of hypertension, 33(3), 465-472.

***Authors’ response:*** *We thank the Editor for the careful and insightful comments regarding our study. We acknowledge that hypertension prevalence, sociodemographic correlates, and broad patterns of inequality in the hypertension care cascade have been previously reported in Bangladesh, including recent analyses based on earlier BDHS rounds and the 2022 BDHS descriptive report.*

*However, we would like to clarify that the primary contribution of our study is not the descriptive replication of known associations, but rather the systematic and comparative quantification of socioeconomic inequalities across the full hypertension care cascade using formal inequality metrics (RII, SII, and Concentration Index) applied to the most recent nationally representative dataset.*

*Specifically, while the BDHS 2022 report provides stratified prevalence and care estimates, it does not quantify inequality gradients using summary indices that capture both relative and absolute socioeconomic gradients across the entire distribution of wealth, nor does it allow comparison of inequality magnitude across different cascade stages (prevalence, diagnosis, treatment, and control) within a unified analytical framework. Similarly, prior published studies in Bangladesh have largely focused on descriptive subgroup comparisons or single measures of inequality, without consistently applying a combined multidimensional inequality framework across all cascade steps in a single study.*

*We simultaneously estimate inequality in prevalence, diagnosis, treatment, and blood pressure control within the same population and analytical framework, allowing direct comparison of where inequities are most pronounced along the cascade. We used complementary, formal inequality measures (RII, SII, and CnI), which allows us to depict both relative and absolute dimensions of inequality, providing a more in-depth understanding than stratified prevalence tables or simple regression outputs. Besides, our findings demonstrate that socioeconomic inequalities are not uniform across the hypertension continuum, with stronger inequalities observed in early cascade stages and attenuation in later stages.*

*We have critically reviewed your suggested articles and strengthened the Introduction and Discussion sections to more explicitly highlight these methodological and interpretive contributions and to clearly differentiate our analysis from prior descriptive reporting of the same dataset.*

*Regarding the second point, we confirm that all analyses were conducted in accordance with DHS analytical guidelines using sampling weights, clustering, and stratification to ensure nationally representative estimates and correct standard errors. This has now been explicitly stated in the Methods section for clarity.*

*We appreciate the opportunity to revise the manuscript and believe that the revised version now more clearly articulates its methodological and analytical contribution beyond existing descriptive reports.*

**Reviewer #1:**

**Title & Abstract**

1. Clearly specify the definition of blood pressure control used (<120/80 mmHg) in the abstract since it differs from standard definitions.

***Authors’ response:*** *We sincerely thank the reviewer for identifying this issue. Following your comment, we carefully rechecked our dataset coding and analytical procedures and confirmed that blood pressure control had actually been defined as <140/90 mmHg, consistent with standard definitions used in previous literature. The previously stated threshold of <120/80 mmHg in the operational definition was an unintended typographical error introduced during manuscript preparation. We have now corrected this definition throughout the manuscript.*

2. Consider including absolute numbers alongside percentages (e.g., number of hypertensive individuals diagnosed/treated) to make the cascade findings more tangible.

***Authors’ response:*** *Thank you for this valuable suggestion. Accordingly, we have included both percentages and corresponding absolute numbers (n/N) for hypertension prevalence, diagnosis, treatment, and blood pressure control in the abstract.*

3. Highlight the novelty with the help of supporting the rationale.

***Authors’ response:*** *Thank you for this suggestion. We have revised the introduction of the abstract accordingly.*

Introduction

4. Shorten the background to reduce redundancy (lines 60–67 repeat known burden data).

***Authors’ response:*** *Thank you for the suggestion. We carefully revised the Introduction section to reduce repetitive discussion regarding the burden of hypertension and streamlined the background to improve clarity and conciseness.*

5. Expand on the knowledge gap — explicitly mention that previous BDHS analyses did not use inequality indices or focused on earlier survey waves.

***Authors’ response:*** *In the revised Introduction, we have now mentioned that most previous studies either relied on earlier survey rounds or primarily presented descriptive estimates without applying formal inequality measures such as RII, SII, or concentration indices. This clarification has been incorporated to better establish the knowledge gap addressed by our study.*

6. Briefly justify why socioeconomic inequalities are critical to measure for policy formulation (universal health coverage, equity monitoring).

***Authors’ response:*** *Thank you for highlighting this important point. We have revised the Introduction to better explain the policy relevance of measuring socioeconomic inequalities, particularly in the context of Bangladesh’s commitment to universal health coverage (UHC), SDG targets, and equity-oriented health system monitoring.*

**Methods**

7. Sampling and Weighting: Explicitly state that sampling weights, clustering, and stratification were incorporated in all descriptive and regression analyses.

***Authors’ response:*** *All analyses were conducted in accordance with DHS analytical guidelines using sampling weights, clustering, and stratification to ensure nationally representative estimates and correct standard errors. This has now been explicitly stated in the Methods section for clarity.*

8. Outcome Definition:

Justify using <120/80 mmHg for blood pressure control (most studies use <140/90 mmHg).

Consider providing sensitivity analysis results with <140/90 mmHg cutoff, at least in supplementary material.

***Authors’ response:*** *We sincerely thank the reviewer for identifying this inconsistency. After rechecking the analytical dataset and coding procedures, we confirmed that blood pressure control had actually been defined using the standard threshold of <140/90 mmHg among treated individuals. The mention of <120/80 mmHg in the manuscript was an inadvertent typographical error. We have now corrected this issue consistently throughout the manuscript. Therefore, additional sensitivity analysis was not necessary, as the original analyses were already conducted using the standard <140/90 mmHg definition.*

Independent Variables: Acknowledge missing key enablers such as health insurance, distance to facility, provider density — discuss how this might bias inequality estimates.

***Authors’ response:*** *Thank you for this important observation. We have now acknowledged these in the Limitations section.*

9. Statistical Analysis:

Mention whether goodness-of-fit tests (e.g., Hosmer–Lemeshow) were conducted for logistic regression.

***Authors’ response:*** *Thank you for the suggestion. We have now added that model fit was assessed for all logistic regression models using the Hosmer–Lemeshow goodness-of-fit test, and all models demonstrated acceptable fit based on non-significant test results (p >0.05).*

Clarify bootstrap procedure for CnI, SII, RII (number of iterations already stated—good).

***Authors’ response:*** *Additional details regarding the bootstrap resampling procedure, including estimation approach and derivation of confidence intervals for inequality measures, have now been added to the Methods section.*

10. Ethics: Include ethical clearance information (BDHS usually has approval from ICF and local authorities, but should be restated for completeness).

***Authors’ response:*** *Thank you for pointing this out. We have now added a detailed ethics statement clarifying that the BDHS 2022 received ethical approval from the relevant national authorities and ICF Institutional Review Board, and that the present study involved secondary analysis of anonymized publicly available data.*

**Results**

11. Include absolute counts in the care cascade (Figure 1) to complement percentages.

***Authors’ response:*** *We appreciate the reviewer’s suggestion. We carefully considered incorporating absolute counts within Figure 1; however, considering the multiple cascade stages and subgroup information presented, inclusion of additional numerical labels substantially reduced figure clarity and readability. To balance interpretability and visual clarity, we retained the current figure format while adding corresponding absolute counts within the tables.*

12. Clarify denominators in Tables 2–4 (e.g., specify n used for each cascade step).

***Authors’ response:*** *Thank you for the suggestion. We have now clarified the denominators used for each cascade stage in the relevant tables to improve interpretability.*

13. Where confidence intervals are very wide (e.g., >70 age group), discuss potential small sample size and interpret with caution.

***Authors’ response:*** *We have now acknowledged in the Limitations section that some subgroup analyses yielded wider confidence intervals, potentially due to smaller sample sizes in specific categories, and that these findings should therefore be interpreted with caution.*

**Discussion**

14. Interpretation of Inequalities: Deepen the interpretation of RII/SII — discuss whether the observed pro-rich distribution is narrowing or widening over time, implying epidemiologic transition.

***Authors’ response:*** *Thank you for this insightful suggestion. We have expanded the Discussion section to provide a more detailed interpretation of the observed inequality measures across prevalence and cascade stages. The revised discussion now contextualizes the pro-rich distribution within the broader epidemiological transition framework and elaborates on how inequalities differ across prevalence, diagnosis, treatment, and control.*

15. Gender & Rural–Urban Findings:

Discuss why women are more likely to be diagnosed/treated but not controlled — possible adherence issues, therapeutic inertia?

***Authors’ response:*** *We appreciate this important comment. We have expanded the Discussion section to explore potential explanations for this pattern, including greater healthcare contact among women, differences in long-term adherence, continuity of care, treatment optimization, and possible therapeutic inertia.*

16. Explore reasons for rural disadvantage and whether it is due to facility distribution or health literacy gaps.

***Authors’ response:*** *Thank you for the suggestion. Additional discussion has now been incorporated regarding possible contributors to rural disadvantage, including differences in healthcare facility availability, diagnostic access, health literacy, continuity of care, and barriers related to healthcare utilization.*

17. Policy Implications: Be more specific — suggest equity monitoring indicators in national hypertension control programs, community-based screening in rural areas, and subsidies for antihypertensive drugs.

***Authors’ response:*** *We appreciate this constructive recommendation. The Discussion section have now been revised to include more specific policy implications, including incorporation of equity-sensitive monitoring indicators within national hypertension programs, strengthening community-based screening initiatives, particularly in underserved areas, and improving affordability and continuity of antihypertensive treatment.*

18. Limitations:

Emphasize potential measurement bias due to cross-sectional BP measurement and recall bias in diagnosis/treatment.

***Authors’ response:*** *Thank you for this important observation. We have expanded the Limitations section to acknowledge the possibility of measurement bias arising from cross-sectional blood pressure assessment, as well as recall bias related to self-reported diagnosis and treatment information.*

Mention the potential effect of using a more stringent BP control definition on prevalence estimates.

***Authors’ response:*** *As noted earlier, the previously reported <120/80 mmHg threshold was a typographical error. All analyses were conducted using the standard <140/90 mmHg definition; therefore, this issue has now been corrected throughout the manuscript.*

**Conclusion:**

19. Reiterate the need for targeted interventions addressing diagnosis and treatment gaps among the poorest quintiles.

***Authors’ response:*** *Thank you for the suggestion. We have revised the Conclusion section to more explicitly emphasize the need for targeted interventions aimed at reducing diagnosis and treatment gaps among socioeconomically disadvantaged populations.*

20. Suggest monitoring equity over time as a key performance indicator for Bangladesh’s hypertension control initiative.

***Authors’ response:*** *We appreciate this valuable recommendation. The Conclusion section has now been expanded to highlight the importance of routine monitoring of socioeconomic inequalities as a key performance indicator within national hypertension control initiatives and broader UHC monitoring frameworks.*

*We sincerely thank the reviewer for the insightful and constructive comments, which, we believe, greatly helped us improve the quality and clarity of the manuscript.*

**Reviewer #2:**

Overall, the manuscript is well drafted. A few questions may arise regarding the following:

1. As the study investigates the socio-economic inequalities in hypertension prevalence and care, why was diabetes prevalence included in the analysis? Please check, if you exclude the diabetes factors and keep only the socio-economic factors, what will the findings be for the logistic regression model?

***Authors’ response:*** *We thank the reviewer for this important comment. T2DM was included as a covariate in the multivariable models based on its established clinical and epidemiological relationship with hypertension and its potential role as a confounding factor in the hypertension care cascade. Our objective was not to assess diabetes prevalence itself, but rather to adjust for important health-related factors that may influence hypertension prevalence, diagnosis, treatment, and control. Since the primary focus of our regression analyses was to identify factors independently associated with hypertension and its care cascade, we retained T2DM in the adjusted models.*

2. A brief note can be added below Table 3. For example, what does the asterisk sign mean? Same for Table 4, the full form of the abbreviation can be added.

***Authors’ response:*** *Thank you for the suggestion. We have now added appropriate table footnotes explaining the meaning of the asterisk symbols and provided the full forms of all abbreviations used in Tables 3 and 4 for improved clarity and readability.*

3. Data presentation and interpretation of Table 4 could be clearer.

***Authors’ response:*** *We appreciate this helpful suggestion. Table 4 has now been revised to improve clarity and interpretability, including standardized decimal formatting, clearer presentation of confidence intervals, and improved labeling of inequality measures and subgroup estimates.*

*We sincerely thank the reviewer for the valuable suggestions, which helped improve the clarity and presentation of our manuscript.*

With best regards

Dr. Md. Golam Hossain

Professor of Health Research Group

Department of Statistics, University of Rajshahi

Rajshahi-6205, Bangladesh

E-mail:hossain95@yahoo.com
