# Supplementary material for: Socio-economic inequalities in hypertension prevalence and care cascade in Bangladesh: Insights from a nationally representative survey
Source: PLOS Glob Public Health. 2026 Jun 23;6(6):e0006712. doi: 10.1371/journal.pgph.0006712 (PMC13289857; doi:10.1371/journal.pgph.0006712)
Supplement: Peer review history

# Peer review: Decision Letter 0

**DOI:** 10.1371/journal.pgph.0006712.r001

**Authors:** Biplab Datta

12 Feb 2026

PGPH-D-25-02335

Socio-economic inequalities in hypertension prevalence and care cascade in Bangladesh: insights from a nationally representative survey

PLOS Global Public Health

Dear Dr. Hossain,

Thank you for submitting your manuscript to PLOS Global Public Health. After careful consideration, we feel that it has merit but does not fully meet PLOS Global Public Health’s publication criteria as it currently stands. Therefore, we invite you to submit a revised version of the manuscript that addresses the points raised during the review process.

Please carefully address the comments by editor and reviewers. Especially, it is needed to establish why your study is a novel investigation and the study is not reporting already known facts on the topic (i.e., sociodemographic inequities in hypertension prevalence and care cascade in Bangladesh).

Please submit your revised manuscript by Mar 29 2026 11:59PM. If you will need more time than this to complete your revisions, please reply to this message or contact the journal office at globalpubhealth@plos.org. When you're ready to submit your revision, log on to https://www.editorialmanager.com/pgph/ and select the 'Submissions Needing Revision' folder to locate your manuscript file.

Please include the following items when submitting your revised manuscript:

- A letter that responds to each point raised by the editor and reviewer(s). You should upload this letter as a separate file labeled 'Response to Reviewers'.
- A marked-up copy of your manuscript that highlights changes made to the original version. You should upload this as a separate file labeled 'Revised Manuscript with Track Changes'.
- An unmarked version of your revised paper without tracked changes. You should upload this as a separate file labeled 'Manuscript'.

Guidelines for resubmitting your figure files are available below the reviewer comments at the end of this letter.

We look forward to receiving your revised manuscript.

Kind regards,

Biplab Datta, Ph.D.

Academic Editor

PLOS Global Public Health

Journal Requirements:

1. Please send a completed 'Competing Interests' statement, including any COIs declared by your co-authors. If you have no competing interests to declare, please state "The authors have declared that no competing interests exist".

1. Please amend your detailed Financial Disclosure statement. This is published with the article. It must therefore be completed in full sentences and contain the exact wording you wish to be published.
  If you did not receive any funding for this study, please simply state: “The authors received no specific funding for this work.”

1. Please upload all main figures as separate Figure files in .tif or .eps format only. For more information about how to convert and format your figure files please see our guidelines:
https://journals.plos.org/globalpublichealth/s/figures

4. If the reviewer comments include a recommendation to cite specific previously published works, please review and evaluate these publications to determine whether they are relevant and should be cited. There is no requirement to cite               these works unless the editor has indicated otherwise.

Additional Editor Comments (if provided):

Hypertension care cascade, especially using DHS data, is a widely studied topic. Previous studies have shown socioeconomic differences in hypertension prevalence (Hossain et al., 2025; Iqbal et al., 2021) and care cascade in Bangladesh (Babagoli et al., 2025; Ishaq & Kakoly, 2025; Rahman et al., 2015). All these studies reported socioeconomic inequities in hypertension care in Bangladesh. As such, what this study is adding to our understanding? Please carefully motivate your work. Using new data (i.e., 2022 DHS) to report already known results may not be acceptable.

Also, hypertension prevalence by sociodemographic groups are clearly reported in the 2022 BDHS report Tables 14.3.1 (for women) and 14.3.2 (for men). From these table we clearly observe that prevalence of hypertension is higher among adults who are older, who have BMI at overweight/obese level, and who are from richest households. As such, what new information are you providing in your analysis that is not previous reported and not stated in the 2022 BDHS report? Note that obesity and diabetes are known risk factors of hypertension (https://www.cdc.gov/high-blood-pressure/risk-factors/index.html). The odds of hypertension should by higher among diabetic individuals and individuals with obesity. Hence, there is nothing new in reporting the known facts.

Hypertension care cascade by sociodemographic groups are reported in the 2022 BDHS report Tables 14.2.1 (for women) and 14.2.2 (for men). Estimates reported in these tables clearly shows that hypertension diagnosis was significantly lower in poorer households compared to richer households. Similarly, likelihood of receiving treatment was also relatively lower in poorer households. As such, the socioeconomic inequity has already been documented in previous studies and the 2022 BDHS report. Hence, what is the contribution of the current study?

Please provide convincing arguments that your study is novel, not reporting already known facts, and provide new information which is critical for hypertension management in Bangladesh. The paper may not be considered for publication without substantial evidence of new knowledge presented.

On a technical note, analyzing DHS data requires use of appropriate survey weights entailing sampling frames (https://dhsprogram.com/data/Guide-to-DHS-Statistics/Analyzing_DHS_Data.htm). It is not clear whether this has been followed while conducting statistical analysis. Please make sure that the estimates and 95% confidence intervals are obtained as per the DHS data analysis recommendations.

References

- Hossain, K., Shuvo, T. A., Hosna, A. U., & Dey, D. R. (2025). The Impact of Socioeconomic Inequalities on the Risk of Hypertension in Bangladesh: A Systematic Review and Meta‐Analysis. The Journal of Clinical Hypertension, 27(1), e14957.

- Iqbal, A., Ahsan, K. Z., Jamil, K., Haider, M. M., Khan, S. H., Chakraborty, N., & Streatfield, P. K. (2021). Demographic, socioeconomic, and biological correlates of hypertension in an adult population: evidence from the Bangladesh demographic and health survey 2017–18. BMC Public Health, 21(1), 1229.

- Babagoli, M. A., Chen, Y. H., Chakma, N., Islam, M. S., Naheed, A., & Boettiger, D. C. (2023). Association of socio-demographic characteristics with hypertension awareness, treatment, and control in Bangladesh. Journal of Human Hypertension, 37(11), 993-999.

- Ishaq, F., & Kakoly, I. J. (2025). Decomposition of socioeconomic inequalities of hypertension care utilization: Bangladesh experience. BMC Health Services Research, 25(1), 1486.

- Rahman, M. M., Gilmour, S., Akter, S., Abe, S. K., Saito, E., & Shibuya, K. (2015). Prevalence and control of hypertension in Bangladesh: a multilevel analysis of a nationwide population-based survey. Journal of hypertension, 33(3), 465-472.

[Note: HTML markup is below. Please do not edit.]

Reviewers' comments:

Reviewer's Responses to Questions

**Comments to the Author**

1. Does this manuscript meet PLOS Global Public Health’s publication criteria? Is the manuscript technically sound, and do the data support the conclusions? The manuscript must describe methodologically and ethically rigorous research with conclusions that are appropriately drawn based on the data presented.

Reviewer #1: Partly

Reviewer #2: Yes

**********

2. Has the statistical analysis been performed appropriately and rigorously?

Reviewer #1: Yes

Reviewer #2: Yes

**********

3. Have the authors made all data underlying the findings in their manuscript fully available (please refer to the Data Availability Statement at the start of the manuscript PDF file)?

The PLOS Data policy requires authors to make all data underlying the findings described in their manuscript fully available without restriction, with rare exception. The data should be provided as part of the manuscript or its supporting information, or deposited to a public repository. For example, in addition to summary statistics, the data points behind means, medians and variance measures should be available. If there are restrictions on publicly sharing data—e.g. participant privacy or use of data from a third party—those must be specified.

Reviewer #1: Yes

Reviewer #2: No

**********

4. Is the manuscript presented in an intelligible fashion and written in standard English?

PLOS Global Public Health does not copyedit accepted manuscripts, so the language in submitted articles must be clear, correct, and unambiguous. Any typographical or grammatical errors should be corrected at revision, so please note any specific errors here.

Reviewer #1: Yes

Reviewer #2: Yes

**********

5. Review Comments to the Author

Please use the space provided to explain your answers to the questions above. You may also include additional comments for the author, including concerns about dual publication, research ethics, or publication ethics. (Please upload your review as an attachment if it exceeds 20,000 characters)

Reviewer #1: Title & Abstract

1. Clearly specify the definition of blood pressure control used (<120/80 mmHg) in the abstract since it differs from standard definitions.

2. Consider including absolute numbers alongside percentages (e.g., number of hypertensive individuals diagnosed/treated) to make the cascade findings more tangible.

3. Highlight the novelty with the help of supporting the rationale.

Introduction

4. Shorten the background to reduce redundancy (lines 60–67 repeat known burden data).

5. Expand on the knowledge gap — explicitly mention that previous BDHS analyses did not use inequality indices or focused on earlier survey waves.

6. Briefly justify why socioeconomic inequalities are critical to measure for policy formulation (universal health coverage, equity monitoring).

Methods

7. Sampling and Weighting: Explicitly state that sampling weights, clustering, and stratification were incorporated in all descriptive and regression analyses.

8. Outcome Definition:

Justify using <120/80 mmHg for blood pressure control (most studies use <140/90 mmHg).

Consider providing sensitivity analysis results with <140/90 mmHg cutoff, at least in supplementary material.

Independent Variables: Acknowledge missing key enablers such as health insurance, distance to facility, provider density — discuss how this might bias inequality estimates.

9. Statistical Analysis:

Mention whether goodness-of-fit tests (e.g., Hosmer–Lemeshow) were conducted for logistic regression.

Clarify bootstrap procedure for CnI, SII, RII (number of iterations already stated—good).

10. Ethics: Include ethical clearance information (BDHS usually has approval from ICF and local authorities, but should be restated for completeness).

Results

11. Include absolute counts in the care cascade (Figure 1) to complement percentages.

12. Clarify denominators in Tables 2–4 (e.g., specify n used for each cascade step).

13. Where confidence intervals are very wide (e.g., >70 age group), discuss potential small sample size and interpret with caution.

Discussion

14. Interpretation of Inequalities: Deepen the interpretation of RII/SII — discuss whether the observed pro-rich distribution is narrowing or widening over time, implying epidemiologic transition.

15. Gender & Rural–Urban Findings:

Discuss why women are more likely to be diagnosed/treated but not controlled — possible adherence issues, therapeutic inertia?

16. Explore reasons for rural disadvantage and whether it is due to facility distribution or health literacy gaps.

17. Policy Implications: Be more specific — suggest equity monitoring indicators in national hypertension control programs, community-based screening in rural areas, and subsidies for antihypertensive drugs.

18. Limitations:

Emphasize potential measurement bias due to cross-sectional BP measurement and recall bias in diagnosis/treatment.

Mention the potential effect of using a more stringent BP control definition on prevalence estimates.

Conclusion:

19. Reiterate the need for targeted interventions addressing diagnosis and treatment gaps among the poorest quintiles.

20. Suggest monitoring equity over time as a key performance indicator for Bangladesh’s hypertension control initiative.

Reviewer #2: Overall, the manuscript is well drafted. A few questions may arise regarding the following:

1. As the study investigates the socio-economic inequalities in hypertension prevalence and care, why was diabetes prevalence included in the analysis? Please check, if you exclude the diabetes factors and keep only the socio-economic factors, what will the findings be for the logistic regression model?

2. A brief note can be added below Table 3. For example, what does the asterisk sign mean? Same for Table 4, the full form of the abbreviation can be added.

3. Data presentation and interpretation of Table 4 could be clearer.

**********

6. PLOS authors have the option to publish the peer review history of their article (what does this mean?). If published, this will include your full peer review and any attached files.

**Do you want your identity to be public for this peer review?**  If you choose “no”, your identity will remain anonymous but your review may still be made public.

For information about this choice, including consent withdrawal, please see our Privacy Policy.

Reviewer #1: **Yes:** Shubhanjali Roy

Reviewer #2: No

**********

[NOTE: If reviewer comments were submitted as an attachment file, they will be attached to this email and accessible via the submission site. Please log into your account, locate the manuscript record, and check for the action link "View Attachments". If this link does not appear, there are no attachment files.]

-->Figure Resubmissions:

While revising your submission, we strongly recommend that you use PLOS’s NAAS tool (https://ngplosjournals.pagemajik.ai/artanalysis) to test your figure files. NAAS can convert your figure files to the TIFF file type and meet basic requirements (such as print size, resolution), or provide you with a report on issues that do not meet our requirements and that NAAS cannot fix.

After uploading your figures to PLOS’s NAAS tool - https://ngplosjournals.pagemajik.ai/artanalysis, NAAS will process the files provided and display the results in the "Uploaded Files" section of the page as the processing is complete. If the uploaded figures meet our requirements (or NAAS is able to fix the files to meet our requirements), the figure will be marked as "fixed" above. If NAAS is unable to fix the files, a red "failed" label will appear above. When NAAS has confirmed that the figure files meet our requirements, please download the file via the download option, and include these NAAS processed figure files when submitting your revised manuscript.

# Author comment: Author response to Decision Letter 1

**DOI:** 10.1371/journal.pgph.0006712.r002

22 May 2026

Attachment Submitted filename: Review comments.docx

# Editor report: Decision Letter 1

**DOI:** 10.1371/journal.pgph.0006712.r003

**Authors:** Biplab Datta, Biplab Datta

4 Jun 2026

Socio-economic inequalities in hypertension prevalence and care cascade in Bangladesh: insights from a nationally representative survey

PGPH-D-25-02335R1

Dear Prof. Hossain,

We are pleased to inform you that your manuscript 'Socio-economic inequalities in hypertension prevalence and care cascade in Bangladesh: insights from a nationally representative survey' has been provisionally accepted for publication in PLOS Global Public Health.

Before your manuscript can be formally accepted you will need to complete some formatting changes, which you will receive in a follow up email. A member of our team will be in touch with a set of requests.

Please note that your manuscript will not be scheduled for publication until you have made the required changes, so a swift response is appreciated.

IMPORTANT: The editorial review process is now complete. PLOS will only permit corrections to spelling, formatting or significant scientific errors from this point onwards. Requests for major changes, or any which affect the scientific understanding of your work, will cause delays to the publication date of your manuscript.

If your institution or institutions have a press office, please notify them about your upcoming paper to help maximize its impact. If they'll be preparing press materials, please inform our press team as soon as possible -- no later than 48 hours after receiving the formal acceptance. Your manuscript will remain under strict press embargo until 2 pm Eastern Time on the date of publication. For more information, please contact globalpubhealth@plos.org.

Thank you again for supporting Open Access publishing; we are looking forward to publishing your work in PLOS Global Public Health.

Best regards,

Biplab Datta, Ph.D.

Academic Editor

PLOS Global Public Health

***********************************************************

Thank you for adequately addressing the comments.

Reviewer Comments (if any, and for reference):
